# The use of ^18^F-fluorodeoxyglucose positron emission tomography (^18^F-FDG PET) as a pathway-specific biomarker with AZD8186, a PI3Kβ/δ inhibitor

**DOI:** 10.1186/s13550-016-0220-9

**Published:** 2016-08-11

**Authors:** Juliana Maynard, Sally-Ann Emmas, Francois-Xavier Blé, Hervé Barjat, Emily Lawrie, Urs Hancox, Deborah Oakes, Urszula M. Polanska, Simon T. Barry

**Affiliations:** 1 Personalised Healthcare and Biomarkers, AstraZeneca, Alderley Park, SK10 4TG UK; 2 Oncology IMED, AstraZeneca, Alderley Park, SK10 4TG UK; 3 Drug Safety and Metabolism IMED, AstraZeneca, Alderley Park, SK10 4TG UK

## Abstract

**Background:**

The phosphatidylinositol 3 kinase (PI3K) signalling pathway is frequently altered in human cancer and a promising therapeutic target. AZD8186 (AstraZeneca) is a PI3Kβ/δ inhibitor, currently in phase 1 clinical trials. ^18^F-fluorodeoxyglucose positron emission tomography (^18^F-FDG PET) is often used as a biomarker for inhibitors targeting the PI3K axis because of the association of this pathway with glucose metabolism. In this study, we assessed if ^18^F-FDG PET could be used as a pharmacodynamic marker to monitor PI3Kβ inhibition by AZD8186, and hence have potential as a clinical biomarker of PI3Kβ pathway activation, and for patient selection. ^18^F-FDG PET scans were performed in nude mice bearing 786-0 renal, U87-MG glioma, and BT474C breast xenograft models. Mice were fasted prior to imaging and static ^18^F-FDG PET imaging was performed. Tumour growth was monitored throughout each study, and at the end of the imaging procedure, tumours were taken and a full pharmacodynamic analysis performed.

**Results:**

Results showed that in PTEN null tumour xenograft models, 786-0 and U87-MG, the PI3Kβ inhibitor AZD8186 reduces ^18^F-FDG uptake at a dose of 50 mg/kg, the same dose which causes tumour inhibition, while it has no impact in a PI3Kα mutant tumour xenograft BT474C. Consistent with the change in ^18^F-FDG uptake, AZD8186 also modulated AKT and associated glucose pathway biomarkers in the PTEN null tumour xenografts but not in PTEN wild-type tumours.

**Conclusions:**

Our pre-clinical studies support the use of ^18^F-FDG PET imaging as a sensitive and non-invasive pharmacodynamic biomarker for use in clinical studies with AZD8186.

**Electronic supplementary material:**

The online version of this article (doi:10.1186/s13550-016-0220-9) contains supplementary material, which is available to authorized users.

## Background

The phosphatidyl inositol 3 kinase (PI3K) signalling pathway is frequently altered in human cancer and is considered a promising therapeutic target to control tumour growth [1]. Normal PI3K-mediated signalling plays a key role in the modulation of cell proliferation, cell survival and metabolism [2]. The PI3K pathway activation can be enhanced through mutation of PIK3CA or AKT, loss of PTEN or amplification of receptor tyrosine kinases, such as HER2 [3], all of which can contribute to tumour progression.

PI3Ks are grouped into three classes based on their substrate specificity and structural features (class 1, 2 and 3) [4]. Class 1 PI3Ks are mutated in many cancers. They primarily phosphorylate phosphatidylinositol-4-5-diphosphate (PIP2) to produce phosphatidylinositol-3-4-5 triphosphate (PIP3). The tumour suppressor gene PTEN reverses this process, and loss of PTEN is associated with upregulation of basal PIP3 generation [5]. PIP3 is required for the activation of the serine threonine kinase, AKT, triggering critical pathways involved in metabolism, cell growth, proliferation, motility and survival [4].

Class 1 PI3Ks are further divided into class 1A (PI3Kα, β and δ) and class 1B enzymes (PI3Kγ) [3]. Many inhibitors have been developed targeting PI3K signalling. The first-generation PI3K inhibitors targeted all PI3K isoforms, but have been limited by toxicity due to the broad selectivity profile. More recently, isoform-selective PI3K inhibitors have been developed to target specific mutations or isoform dependencies in different tumour types [3]. These more selective inhibitors reduce some of the dose-limiting side effects that made the first generation of less selective agents less successful than first anticipated [6]. The PI3Kα subtype of PI3K receptor has been the most extensively studied to date and various PI3Kα inhibitors exist which are currently undergoing clinical trials [7]. Inhibitors targeting other PI3K isoforms have also been developed. Indeed, the first PI3K inhibitor to gain approval for treatment of chronic lymphoid leukaemia was GS-1101 (Idelalisib) (Gilead Sciences), which is an inhibitor of PI3Kδ. A second class of isoform-selective inhibitors that have been developed are those targeting PI3Kβ. Deletion of the tumour suppression PTEN can confer sensitivity for inhibitors targeting the PI3Kβ isoform [8]. A number of PI3Kβ inhibitors TGX-221 [9], GSK2636771 (GlaxoSmithKline) [10], SAR260310 (Sanofi) [11] and AZD8186 (β/δ) (AstraZeneca) [12] are in early clinical trials. So far, pre-clinical data has established the potential of these inhibitors in tumours that have lost PTEN and became dependent on PI3Kβ [13, 14]. To use these inhibitors more effectively, it is essential that biomarkers are developed that enable the potential effectiveness of PI3K isoform-selective inhibitors in an individual tumour to be determined.

While baseline genetic or IHC analysis can identify tumours with an appropriate genetic profile, e.g. PI3Kα mutation, PTEN mutation or loss, it is more challenging to determine whether pathway activation will be reduced by a particular drug. Commonly, this can be achieved through invasive techniques to obtain a tumour biopsy pre- and post-treatment and then assess changes in phospho-biomarkers. In the clinic, this is challenging to deliver, it creates a burden on the patient and performing the analysis takes time. To address this limitation, we have considered whether non-invasive techniques can enable a patient’s tumour(s) to be screened for PI3K isoform dependency to guide effective selection of patients for therapy [15].

^18^F-fluorodeoxyglucose positron emission tomography (^18^F-FDG PET) is often used as a pharmacodynamic biomarker with inhibitors targeting the PI3K axis because of the association of this pathway with glucose metabolism [16]. ^18^F-FDG PET has been commonly used to assess agents in the PI3K pathway (largely PI3Kα, mTOR and AKT) both clinically [17] and pre-clinically [16] and as a biomarker for therapeutic response [18, 19]. In this study, we assessed whether ^18^F-FDG PET could be used as a pharmacodynamic marker to monitor PI3K inhibition by AZD8186.

To assess ^18^F-FDG PET as a biomarker for AZD8186 in population enriched for PTEN deficiency, we measured ^18^F-FDG uptake and AKT pathway activity biomarkers in PTEN null (786-0 renal and U87-MG glioma in vivo models) and compared it to the results acquired in a PTEN-proficient BT474C breast cancer xenografts.

## Methods

### Animal model

Cell lines used had the following mutations associated with the PI3K pathway; 786-0 were PTEN-null, WT for PI3Kα and PI3Kβ isoforms. U87-MG were PTEN-null and WT for PI3Kα and PI3Kβ isoforms. BT474C have WT PTEN and PI3Kβ, but carry mutation in PIK3CA/PI3Kα (K111N) which renders the tumour sensitive to PI3K alpha inhibitors [20, 21].

Female nude (nu/nu:Alpk) or SCID mice (Harlan, UK) were used and maintained in rooms under controlled conditions of temperature (19–23 °C) and humidity (55 % ± 10 %), photoperiod (12 h light/12 h dark) and air exchange, with food and water provided ad libitum. The facilities have been approved by the Home Office and meet all current regulations and standards of the UK. Mice underwent subcutaneous inoculation of the following human tumour cell lines: 786-0 renal, U87-MG glioblastoma and BT474C breast carcinoma. Cells were implanted on the left flank in a volume of 0.1 ml in RPMI medium containing 1.0 × 10^6^ cells per mouse for the U87-MG cell line (no Matrigel) and 5.0 × 10^6^ cells per mouse for the BT474C and 786-0 cell lines (with Matrigel from Beckton Dickinson). For BT474C studies, the animals were supplemented with 0.36 mg/60 day 17-β-estradiol pellets (Innovative Research of America) 1 day prior to cell implantation. U87-MG cells were grown in MEM media with added 10 % FCS, 1 % glutamine, 1 % non-essential amino acids, and 1 % pyruvate. BT474C were grown in DMEM supplemented with 10 % FCS, 1 % glutamine, 1 % oxaloacetic acid, and 10 % M1 (EGG technologies); 786-0 cells were cultured in RPMI supplemented with 10 % FCS, 1 % glutamine, 1 % Na pyruvate and 10 mM HEPES, and 2.5 g/l. Unless stated otherwise, media and all additives were sourced from Sigma.

For in vivo implant, cells were grown to 80–90 % confluency and harvested from T225 tissue culture flasks after treatment with 0.05 % trypsin (Invitrogen) in EDTA solution followed by a suspension in basic medium and three washes in phosphate-buffered saline (Invitrogen). Only single-cell suspensions of viability greater than 90 %, as determined by trypan blue exclusion, were used for injection.

### Efficacy and imaging studies

For growth curves, mice were dosed twice daily with AZD8186 (50 mg/kg) and measured twice weekly using Vernier callipers. Growth inhibition from the start of treatment was assessed by comparison of the geometric mean change in tumour volume for the control and treated groups.

When mean tumour sizes reached approximately 0.2 cm^3^, mice were randomized into control and treatment groups. Tumours were measured using bilateral Vernier calliper measurements using the formula (length × width) × √(length × width) × (π/6).

Treatment groups received 50 mg/kg AZD8186 or vehicle (80 % Tween) at a dose volume of 10 ml/kg.

On the day of imaging, food was withdrawn at 7 a.m. so mice were fasted at least four hours prior to imaging and dosed 2 hours prior to imaging. Blood glucose concentration was measured before vehicle or drug administration and after PET scanning. Blood glucose concentrations were measured using an AccuChek metre (AVIVA, Roche). Anaesthesia was induced using isoflurane delivered in 100 % oxygen (~1.5 % isoflurane, 3 L oxygen). Respiration and temperature were maintained throughout, with body temperatures being maintained at 36–37 °C.

### ^18^F-FDG PET imaging

^18^F-FDG was supplied by PETNET solutions, Nottingham, UK. Imaging was performed using the Inveon multimodality™ PET scanner from Siemens Medical Solutions.

Mice received approximately 15 MBq ^18^F-FDG administered as an i.v. bolus. The % injected dose of ^18^F-FDG that entered the animal was calculated using a BriTec well counter and a measurement performed before and after ^18^F-FDG injection. Following ^18^F-FDG uptake, anaesthesia was maintained for a 45-min uptake period following by a 20-min emission PET scan. Data were acquired using Inveon Acquisition Workplace (IAW) software (Siemens) version 2.2.0. The performance of the scanner has been documented previously [22]. Images were reconstructed using the order subset expectation maximisation (OSEM)/maximum a posterior (MAP) algorithm. Regions of interest (ROIs) were drawn manually using the 3D visualization package of IRW software. Data were expressed as the maximum standardized uptake value (SUVmax). SUVmax was calculated using the formula described by Gambhir et al. [23].

### Biodistribution analysis

At the end of scanning, mice were humanely euthanized and blood, muscle, lung, liver, tumour, bone and tail samples collected. Samples were weighed, and weights were recorded. Tissue samples were counted in a gamma counter (Perkin Elmer, 1480, Wizard 3) for 20 s per sample. The gamma counter provides a “counts per minute” parameter. Data were imported into an Excel spreadsheet, and the counts per minute were converted into activity by conversions into disintegrations per minute; by multiplying the efficiency of the gamma counter for ^18^F. Activity was decay corrected to the time of injection and converted into a concentration using the ex vivo tissue weight (in kilobecquerel per gramme). All mice in which the tail activity exceeded 10 % of the injected dose were excluded from analysis.

### Pharmacodynamic analysis

Following removal, tumours were cut in half. Half was placed in 4 % buffered formalin for 24 h before being stored in 70 % ethanol. Tumours were processed in wax and immuno-histochemical analysis performed. Quantification of phospho-AKT and phopsho-PRAS40 expression was determined. Slides were incubated with rabbit Mab anti-phospho PRAS40 (Thr 246) (CD77D7; dilution 1:200) for detecting pPRAS40 expression and rabbit Mab anti-human phospho AKT-(Ser 473) (Dako M3628; dilution 1:50) for assessing the level of AKT activation. Detection of immunostaining was performed using a Dako Rabbit Envision HRP polymer kit (Dako, Cat. #51699).

The remaining half of the tumour was snap frozen in liquid nitrogen and stored at −80 °C.

Lysates were generated as follows: 0.8 mL of ice-cold lysis buffer (Invitrogen, FNN0011), supplemented with phosphatase inhibitors 2 and 3: Sigma #P0044, Sigma #P5726 (diluted 1:100) and protease inhibitors: Sigma #P8340 (diluted 1:200), was added to each tumour in a Fastprep tube (MP Biomedicals #6910-500). The tumours were homogenized twice for 1 min on a MP Biomedicals Fast Prep-24 machine at 6.5 m/s and then left on ice for 5 and 15 min (following the first and second disaggregation cycles, respectively). The protein concentrations were measured in cleared lysates using BCA protein Assay kit (Thermo Fisher Scientific, 23228 and 233224) prior to all pharmacodynamic assays.

786-0 frozen tumours were used for MesoScale Discovery and ELISA assay analysis. Quantification of AKT phosphorylated at Ser473 was performed using 50 μg of each protein extract diluted in freshly prepared lysis buffer in a 96-well plate and used for MesoScale Discovery (MSD) Assay (Whole Cell Lysate Kit, K15100D-3) according to the manufacturer’s instructions and developed using SECTOR Imager. Analysis and quantification of PRAS40 was performed by sandwich ELISA as per manufacturer’s instructions (Life Technologies # KHO0421). All additional pharmocodynamic biomarker data presented for 786-0 and all data for U87-MG and BT474C tumours were generated by Western blot using following antibodies: phospho-AKT Ser473 (CST, #4060, 1:1000), phospho-PRAS40 Thr246 (CST, #13175, 1:1000), CC3 (Abcam, ab32042, 1:500), yH2AX Ser139 (Millipore, 05-636, 1:500), phospho-AS160 Thr642 (CST, #8881, 1:500), Glut-4 (Abcam, ab65267, 1:1000), and vinculin as a loading control (Abcam, ab18058, 1:5000).

For each Western blot analysis, 45 μg of protein was run. Samples containing1× NuPAGE LDS sample buffer (Invitrogen NP0008) and Invitrogen reducing agent (NP0004) were boiled for 5 min at 95 °C and loaded on 1.0-mm-thick BisTris 4–12 % gradient gels (Invitrogen#NP0323BOX). Gels were run for 60 min at 180 V in 1× NUPAGE MES SDS running buffer (Invitrogen #NP0002) in the presence of NuPAGE antioxidant (Invitrogen #NP0005). Proteins were electro-transferred to 0.2-μm nitrocellulose membranes (Invitrogen # IB3010-01) using an Iblot dry blotting system (Invitrogen #IB1000 UK). After blocking with a 5 % non-fat dried milk in Tris-buffered saline solution containing 0.05 % Tween (TBST), the membranes were probed O/N with primary antibodies and then washed with TBST (3 × 15 min) and probed with HRP-conjugated secondary antibodies for 1 h. Binding was visualized using SuperSignal West Dura Chemiluminescent Substrate reagent (Pierce Thermo Scientific). Biomarker signals were quantified using Genetools software and normalised to vinculin used as a loading control. A two sided *t* test was performed on data assuming unequal variance. Vehicle controls were used for normalizing biomarker signal for the treated samples.

### Statistical analysis for imaging studies

Data are reported as the mean ± SEM unless otherwise stated. Statistical analyses were performed using Graph Pad Prism (v4.02) and group means compared using a two-sided *t* test. The significance level was set to *p* < 0.05.

## Results

### AZD8186 inhibits growth of PTEN-null but not PIK3CA/PI3Kα mutant tumour xenografts

**Fig. 1:**
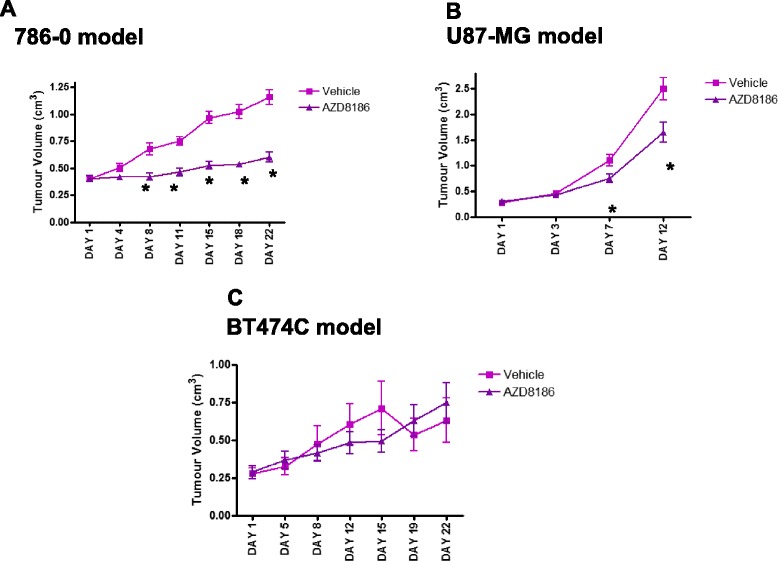
A single dose of AZD8186 (50 mg/kg) shows tumour growth inhibition in PTEN mutated models of cancer with selectivity against PI3Kα. Tumour volume following AZD8186 dosing (mean ± SEM). **a** 786-0: vehicle *n* = 10; AZD8186 *n* = 10. **b** U87-MG: vehicle *n* = 12; AZD8186 *n* = 9. **c** BT474C: vehicle *n* = 12; AZD87186 *n* = 9. *p < 0.05 unpaired *t* test

Consistent with the hypothesis that PI3Kβ has an important role in promoting the growth of PTEN-null tumours, AZD8186 inhibited the growth of the PTEN-null tumour xenograft models 786-0 (renal cell line) and U87-MG (glioma), but did not inhibit the PIK3CA/PI3Kα mutant breast tumour xenograft model BT474C (Fig. 1a–c).

The 50 mg/kg AZD8186 inhibited the growth of the 786-0 and U87-MG PTEN null cell lines. The 786-0 xenograft model showed a significant 74 % inhibition in tumour growth by the end of the study (*p* < 0.0001) in the AZD8186-treated group compared to vehicle (Fig. 1a). A significant inhibition in tumour growth was also seen in the U87-MG xenograft model with a 42 % reduction in tumour growth (*p* = 0.001) achieved at the end of the study (Fig. 1b). No efficacy was seen in the BT474C model where a 17 % increase in tumour growth was observed at the end of the study between the vehicle and AZD8186-treated group (*p* > 0.05) (Fig. 1c).

### ^18^F-FDG PET is a pathway-specific biomarker for AZD8186

The potential of ^18^F-FDG PET as a pharmacodynamic biomarker for AZD8186 was measured by its ability to reduce ^18^F-FDG uptake after a single dose.

**Fig. 2:**
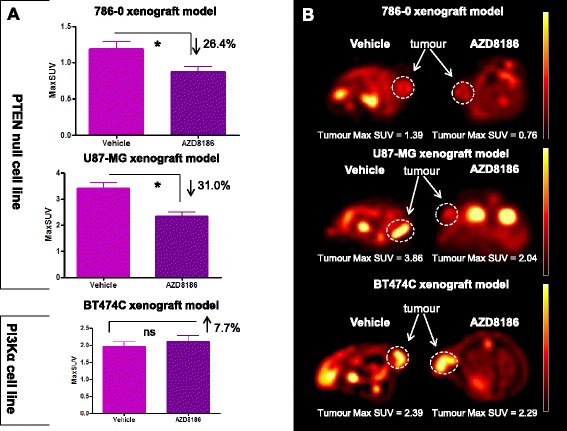
A single dose of AZD8186 (50 mg/kg) reduces 18F-FDG uptake 2 h after dosing in the PTEN null 786-0 and U87-MG xenograft models but not the BT474C PI3Kα model. **a** MaxSUV tumour 18F-FDG uptake following AZD8186 dosing (mean ± SEM). **b** Representative images from each cell line. 786-0: vehicle *n* = 10; AZD8186 *n* = 11. U87-MG: vehicle *n* = 8; AZD8186 *n* = 9. BT474C: vehicle *n* = 7; AZD87186 *n* = 8. *p < 0.05 unpaired *t* test

In the PTEN-null 786-0 tumour xenografts, there was a significant decrease in tumour ^18^F-FDG uptake seen following AZD8186 treatment, with a 26 % reduction (*p* = 0.007) in ^18^F-FDG uptake observed compared to the vehicle treated group (Fig. 2). Tumour SUVmax from imaging data was 1.19 ± 0.09 and 0.87 ± 0.07 in the vehicle and AZD8186-treated groups, respectively.

A significant reduction in ^18^F-FDG uptake following administration of AZD8186 was also observed post-treatment in the U87-MG tumour xenografts. In the U87-MG model, a 31 % decrease (*p* = 0.0004) in ^18^F-FDG uptake was observed in the AZD8186-treated group compared to vehicle (Fig. 2). Tumour SUVmax from imaging data was 3.41 ± 0.20 and 2.33 ± 0.16 in the vehicle and AZD8186-treated groups, respectively.

No significant change in ^18^F-FDG uptake following administration of AZD8186 was observed post-treatment in the PI3Kα-dependent BT474C tumour xenografts (Fig. 2). Tumour SUVmax from imaging data was 1.95 ± 0.14 and 2.10 ± 0.19 in the vehicle and AZD8186-treated groups, respectively.

### ^18^F-FDG PET showed tumour specific modulation and no systemic glucose changes following AZD8186 administration

PI3Kβ inhibitors are differentiated from PI3Kα, mTOR and AKT inhibitors because they do not modulate systemic glucose and other aspects of the canonical PI3Kα signalling pathway [2]. This makes them very attractive as therapeutic agents as they do not exhibit the systemic off-target effects of other PI3K, mTOR and AKT inhibitors and also makes them attractive candidates for combination partners with other PI3K inhibitors.

To assess the specificity of ^18^F-FDG PET as a pharmacodynamic biomarker, changes in ^18^F-FDG uptake were assessed in background tissues following AZD8186 administration.

**Fig. 3:**
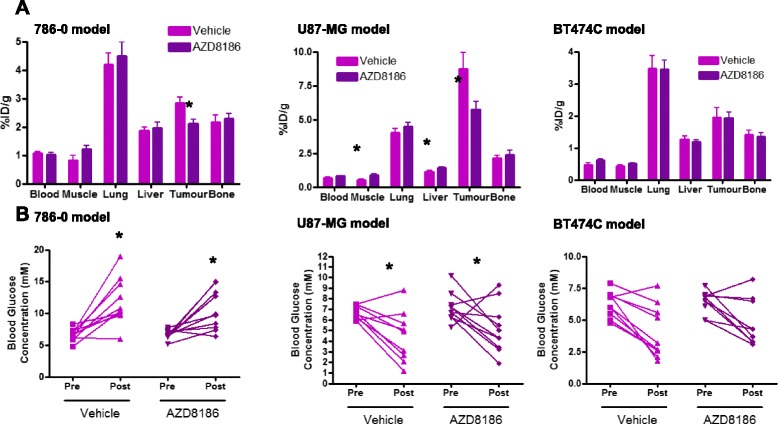
A single dose of AZD8186 (50 mg/kg) shows no systemic glucose changes and tumour specific modulation 2 h after dosing. **a** %ID/g biodistribution data in the blood, muscle, lung, liver tumour and bone (mean ± SEM). **b** Blood glucose concentration pre- and post-dosing (individual animal data). 786-0: vehicle *n* = 10; AZD8186 *n* = 10. U87-MG: vehicle *n* = 8; AZD8186 *n* = 9. BT474C: vehicle *n* = 7; AZD87186 *n* = 8. One-way ANOVA 786-0 *p* < 0.0001; U87-MG *p* = 0.0017; BT474C *p* = 0.002. Student-Newman-Keuls post hoc analysis performed with *p < 0.05 pre- vs post #*p* < 0.05 vs treatment

In the 786-0 PTEN-null model, biodistribution data showed that there were no significant changes in any of the peripheral tissues counted and that there was tumour-specific modulation of ^18^F-FDG uptake only (Fig. 3a). Consistent with the imaging data, a 25 % reduction in tumour uptake was observed post AZD8186 treatment. Blood glucose measured before and after treatment in the 786-0-treated group also showed no significant change in blood glucose concentration following drug treatment. There was a significant increase in blood glucose concentration between both the vehicle and AZD8186 treated group pre- and post-treatment. We have observed this response previously in treated SCID mice associated with anaesthesia and/or fasting (unpublished data) (Fig. 3b). Blood glucose concentration was 6.4 mM ± 0.24 (pre-) and 12.1 mM ± 0.98 (post-) in the vehicle-treated group and 6.6 mM ± 0.21 (pre-) and 10.7 mM ± 0.93 (post-) in the AZD8186-treated group.

In the U87-MG cell line, there was a significant increase in ^18^F-FDG uptake in the muscle and liver in the AZD8186-treated group compared to vehicle (a 70 and 25 % increase, respectively (Fig. 3a). %ID/g in the muscle was 0.52 ± 0.06 and 0.88 ± 0.11 in the vehicle and AZD8186-treated groups, respectively, and % ID/g in the liver was 1.12 ± 0.10 and 1.41 ± 0.08 in the vehicle and AZD8186-treated groups, respectively. Consistent with the imaging data, there was also a 34 % decrease in ^18^F-FDG uptake in the tumour in the AZD8186-treated group compared to vehicle (Fig. 3a). There were no significant changes in any other tissues. Blood glucose concentration also showed no significant changes between the vehicle and AZD8186-treated group (Fig. 3b). Blood glucose concentration was 6.7 ± 0.19 (pre-) and 4.5 ± 0.76 (post-) in the vehicle-treated group and 7.2 ± 0.43 (pre-) and 5.2 ± 0.73 (post-) in the AZD8186-treated group.

In the BT474C cell line, there were no significant changes in any background tissues including the tumour, in concordance with the imaging data (Fig. 3a). Blood glucose concentration also showed no significant changes between the vehicle and AZD8186-treated group in this cell line (Fig. 3b). Blood glucose concentration was 6.3 ± 0.31 (pre-) and 4.0 ± 0.65 (post-) in the vehicle-treated group and 6.4 ± 0.27 (pre-) and 4.8 ± 0.61 (post-) in the AZD8186-treated group.

### ^18^F-FDG PET data correlates with other pharmacodynamic endpoints

Independent analyses of tumour samples by immuno-histochemistry and Western blot confirmed significant suppression of AKT and PRAS40 phosphorylation by AZD8186 (both markers of PI3K pathway activation) in 786-0 and U87-MG tumour xenografts that lack functional PTEN. No changes were detected in the PTEN proficient (PI3Kα mutant) tumour xenograft (BT474C).

**Fig. 4:**
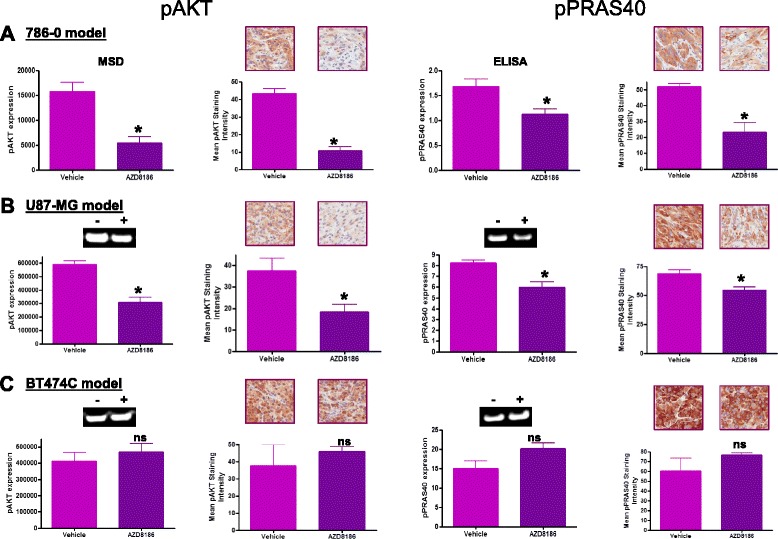
A single dose of AZD8186 (50 mg/kg) shows a pharmacodynamic knock down of PI3K pathway activity in both PTEN null cell line models and not in PI3Kαin correlation with imaging data. **a** 786-0 model. **b** U87-MG model. **c** BT474C model. Data is represented as mean ±SEM. pAKT expression represented by MSD, Western blot and IHC analysis; pPRAS40 expression represented with ELISA, Western blot and IHC analysis. IHC *n* = 5/group. MSD and Western blots *n* > 8/group. Representative images for Western blot (±AZD8186) and IHC analysis (×20 magnification) are also shown

In the 786-0 tumour xenografts, Western blot analysis revealed a significant decrease (66 and a 33 %, *p* < 0.05) in the level of phosphorylated AKT and PRAS40, respectively, in the AZD8186-treated group compared to vehicle (Fig. 4a). Consistent with this immuno-histochemical analysis showed a significant decrease (75 and a 27 %, *p* < 0.05) in the staining intensity of phospho-AKT and phospho-PRAS40 in the AZD8186-treated group compared to vehicle (Fig. 4a).

In the U87-MG tumour xenograft, Western analysis revealed a significant decrease (47 and a 27 %, *p* < 0.05) in the level of phosphorylated AKT and PRAS40 expression, respectively, in the AZD8186-treated group compared to vehicle (Fig. 4b). Consistent with this immuno-histochemical analysis revealed a significant decrease (51 and a 26 %, *p* < 0.05) in the staining intensity of phospho-AKT and phospho-PRAS40 in the AZD8186-treated group compared to vehicle (Fig. 4b).

In the BT474C cell line, Western blot analysis showed no significant change in the level of phospho-AKT and phospho-PRAS40, respectively, in the AZD8186-treated group compared to vehicle (Fig. 4c). Immuno-histochemical analysis indicates no significant change in the staining intensity of phospho-AKT and phospho-PRAS40 in the AZD8186-treated group compared to vehicle (Fig. 4c). Representative blots of all biomakers are shown inset in Fig. 4 and in Additional file 1: Figure S1.

To understand the link between PI3Kβ inhibition, downstream biomarkers and inhibition of glucose uptake, we assessed the changes in phosphorylation of AKT Substrate of 160 kDa (phospho-AS160, Thr642), Glut-4 and Hexokinase 2 (HK2) post AZD8186 treatment. It was not possible to assess expression of Glut1. In 786-0, phospho-AS160 was modulated, while in U87-MG Glut4, expression was reduced following AZD8186 (Additional file 2: Figure S2). Hexokinase 2 was not consistently modulated in the PTEN-null models. No changes were observed in the BT474C tumours (Additional file 2: Figure S2). It is possible that in addition to pathway biomarkers, the ability of AZD8186 to induce cell death could influence glucose uptake. In the PTEN-null tumour models, no robust induction of cleaved caspase 3 was observed; however, changes in γH2AX were seen in 786-0 and to lesser extent in U87-MG. No induction was seen in BT474C (Additional file 3: Figure S3). Collectively, these data suggest that the changes in glucose uptake are a result of acute changes in the activity of the PI3K pathway.

## Discussion

The PI3Kβ isoform is expressed in all cells, but is not a major PI3K isoform driving AKT activation. In normal cells, PI3Kβ plays a critical role in platelets aggregation downstream of GPCRs activation. In tumours, loss of PTEN protein expression creates a dependency on PI3Kβ for cell survival and proliferation [13, 14]. Moreover, PI3Kβ can be mutated (low incidence) or drive signal downstream of mitogenic GPCRs or facilitate the transforming activity downstream of mutant Rac GTPase. PI3Kβ is not thought to play a major role in regulation of glucose uptake in normal tissues.

We have shown that in the PTEN-null tumour xenograft models, 786-0 and U87-MG, the PI3Kβ inhibitor AZD8186 reduces ^18^F-FDG uptake, while it has no impact in a PI3Kα mutant tumour xenograft BT474C. Consistent with the change in ^18^F-FDG uptake, AZD8186 modulated phospho-AKT and associated pathway biomarkers in the PTEN-null tumour xenografts, but not in the PTEN wild-type tumour model in which pan PI3K and PI3Kα family inhibitors are active [12]. In the PTEN-null models, AZD8186 reduced phosphorylation of AS160 (AKT substrate of 160 kDa), an AKT-activity-dependent modulator of Glut transporter expression and localisation. Inhibition of Glut function reduces glucose uptake. We were unable to determine Glut-1 expression in the models; however, Glut-4 expression was suppressed in PTEN-null model U87-MG. In 786-0 tumours, Glut-4 was poorly expressed and levels were not regulated. None of these biomarkers were modulated in the PTEN wild-type BT474C tumours. These data establish the proof of principle that ^18^F-FDG PET could be used as a specific pharmacodynamic biomarker for AZD8186 and other PI3Kβ inhibitors with potential for its use as a clinical biomarker.

Interestingly in both the PTEN-null tumour xenografts tested, there was no difference in the magnitude of reduction in ^18^F-FDG uptake. The reduction in SUVmax uptake (>25 %) with both was the equivalent to a partial metabolic response in clinical practice.^18^F-FDG PET has been commonly used to assess multiple agents in the PI3K pathway because of its role in glucose metabolism. We have previously explored modulation of ^18^F-FDG uptake with an AKT inhibitor AZD5363 [24], a PI3Kα inhibitor AZD8835 (manuscript in preparation) and a mTOR inhibitor AZD8055 [25]. These studies demonstrated the potential for ^18^F-FDG PET to act both as a pharmacodynamic biomarker and a biomarker of response. Both the AKT and PI3Kα inhibitor decrease ^18^F-FDG uptake in the PI3Kα cell line BT474C cell line. The lack of modulation of ^18^F-FDG uptake by AZD8186 in BT474C tumours validates the hypothesis that the effects are due to specific inhibition of PI3Kβ in the PTEN-null tumour xenografts.

The changes detected by imaging correlated with other pharmacodynamic effects of AZD8186 on reduced PI3K pathway biomarkers in the PTEN-null tumour models and not in the PI3Kα mutant model BT474C. Phosphorylation of AKT and PRAS40 both reduced following AZD8186 treatment in PTEN null cell lines, though the change in phospho-AKT was more pronounced than the change in phopsho-PRAS40, an effect seen in other studies [12]. The direct modulation of glucose uptake is suggested by the suppression of AS160 phosphorylation, which regulates Gluts trafficking, localisation or recycling. While we were able to show that Glut-4 expression was suppressed in U87-MG, it would be informative to understand the differential regulation of other Glut family members to gain greater insight in to the determinants of glucose uptake.

To investigate whether acute induction of cell death could impact the ^18^FDG uptake post-AZD8186 treatment in 786-0, U87-MG and BT474C, we looked at cleaved caspase-3 (CC3) as a measure of apoptotic cell death. There was no significant induction of apoptosis implying that cell death does not influence the ^18^FDG uptake over the time course of the study. Changes in the DNA damage in response to cellular stress cell stress was also studied by assessing phosphorylation of H2AX at Ser139 (γH2AX S139). Induction of DNA damage occurred in AZD8186-sensitive models, 786-0 and U87-MG, indicating an induction of a significant level of cellular stress in these tumours, possibly related to the changes in glucose uptake. This is consistent with other observations following monotherapy AZD8186 treatment in other pre-clinical models (unpublished data).

There was no significant difference in blood glucose concentrations following AZD8186 administration at the end of the imaging procedure, supporting the hypothesis that PI3Kβ plays a minor role in normal tissue glucose uptake. The increase in blood glucose was seen between pre- and post-treatment measurements in both the vehicle and AZD8186-treated SCID mice and is a consistent consequence of anaesthesia/fasting in this strain. In contrast other PI3K pathway inhibitors (AKT and PI3Kα) induced an increase in blood glucose relative to vehicle following the imaging procedure (24, manuscript in preparation). It is hypothesized that the increase in blood glucose that is observed following administration of these agents is as a result of altered hepatic glycogen metabolism and blockage of peripheral glucose uptake through Glut4 [26].

In 786-0 and BT474C, the biodistribution data suggests little impact of AZD8186 on normal tissues, and ^18^F-FDG uptake changes in tumour from biodistribution data corresponds with the imaging data and a significant decrease in tumour ^18^F-FDG uptake. The lack of effect in other tissues confirms the hypothesis that PI3Kβ inhibitors such as AZD8186 will show limited effects on normal tissues compared with other pan PI3K inhibitors [12].

Interestingly, biodistribution data showed an increase in ^18^F-FDG uptake in the muscle and liver in the U87-MG cell line. This effect of an increase in ^18^F-FDG uptake in the muscle and liver has also been seen with the PI3Kα inhibitor and AKT in-house (unpublished data) at higher doses. This may be a consequence of altered glycogen metabolism and inhibition of peripheral glucose uptake through Glut4 [26]. The latter was suppressed in U87-MG tumours post AZD8186 treatment. Further work is required to elucidate the mechanism of cross talk between U87-MG tumours and normal tissues.

## Conclusions

In conclusion, our pre-clinical studies support the use of ^18^F-FDG PET imaging as a sensitive and non-invasive pharmacodynamic biomarker for use in clinical studies with AZD8186.

^18^F-FDG PET demonstrated tumour-specific modulation of glucose uptake only following AZD8186 administration with no differences seen in peripheral tissues making it a very effective combination partner for use with other PI3K inhibitors and confirming the differentiating factor of PI3Kβ inhibitors as a whole.

## Additional files

Additional file 1: Figure S1. A single dose of AZD8186 (50 mg/kg) shows a pharmacodynamic knock down of PI3K pathway activity in both PTEN null cell line models and not in PI3Kαin correlation with imaging data—representative readout of AKT pathway biomarkers signal. (PDF 72 kb) Additional file 2: Figure S2. A single dose of AZD8186 (50 mg/kg) results in a non-uniform modulation of biomarkers associated with glucose uptake in different tumour models tested. Phosphorylation of AS160 (Thr642) and expression of Glut-4 and HK2: A) 786-0 model; B) U87-MG model; C) BT474C model. Western blot data is shown for individual animals and geomeanindicated; *n* of at least eight tumours/group. D) Example Western blots for each biomarker in each model without and with AZD8186 treatment. (PDF 76 kb) Additional file 3: Figure S3. A single dose of AZD8186 (50 mg/kg) results in only limited apoptotic cell death and upregulatedcellular stress in PTEN null models. Cleaved caspase-3 (apoptosis) and γH2AX Ser139 levels (DNA damage, stress response): A) 786-0 model; B) U87-MG model; C) BT474C model. Western blot data is shown for individual animals and geomeanindicated; *n* > 8/group. D). Example Western blots for each biomarker in each model without and with AZD8186 treatment. (PDF 166 kb)
