# Supplementary material for: The use of 18F-fluorodeoxyglucose positron emission tomography (18F-FDG PET) as a pathway-specific biomarker with AZD8186, a PI3Kβ/δ inhibitor
Source: EJNMMI Res. 2016 Aug 11;6:62. doi: 10.1186/s13550-016-0220-9 (PMC4980858; doi:10.1186/s13550-016-0220-9)
Supplement: Additional file 1: Figure S1. — A single dose of AZD8186 (50 mg/kg) shows a pharmacodynamic knock down of PI3K pathway activity in both PTEN null cell line models and not in PI3Kαin correlation with imaging data—representative readout of AKT pathway biomarkers signal. (PDF 72 kb) [file 13550_2016_220_MOESM1_ESM.pdf]

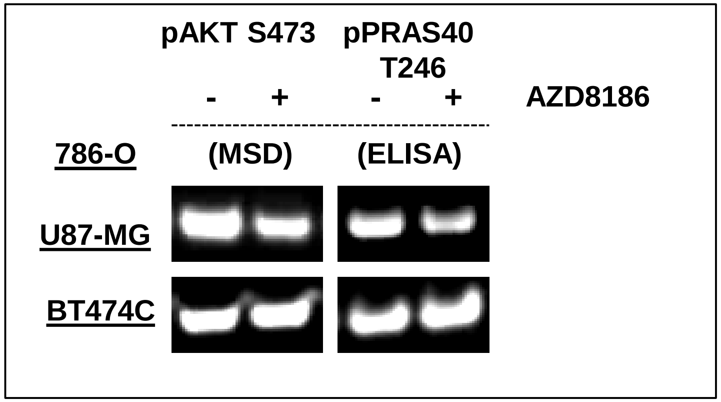


**Supplementary Figure 1: A single dose of AZD8186 (50mg/kg) shows a pharmacodynamic knock down of PI3K pathway activity in both PTEN null cell line models and not in PI3Kα in correlation with imaging data** - representative readout of AKT pathway biomarkers signal.
