# Supplementary material for: The use of 18F-fluorodeoxyglucose positron emission tomography (18F-FDG PET) as a pathway-specific biomarker with AZD8186, a PI3Kβ/δ inhibitor
Source: EJNMMI Res. 2016 Aug 11;6:62. doi: 10.1186/s13550-016-0220-9 (PMC4980858; doi:10.1186/s13550-016-0220-9)
Supplement: Additional file 2: Figure S2. — A single dose of AZD8186 (50 mg/kg) results in a non-uniform modulation of biomarkers associated with glucose uptake in different tumour models tested. Phosphorylation of AS160 (Thr642) and expression of Glut-4 and HK2: A) 786-0 model; B) U87-MG model; C) BT474C model. Western blot data is shown for individual animals and geomeanindicated; n of at least eight tumours/group. D) Example Western blots for each biomarker in each model without and with AZD8186 treatment. (PDF 76 kb) [file 13550_2016_220_MOESM2_ESM.pdf]

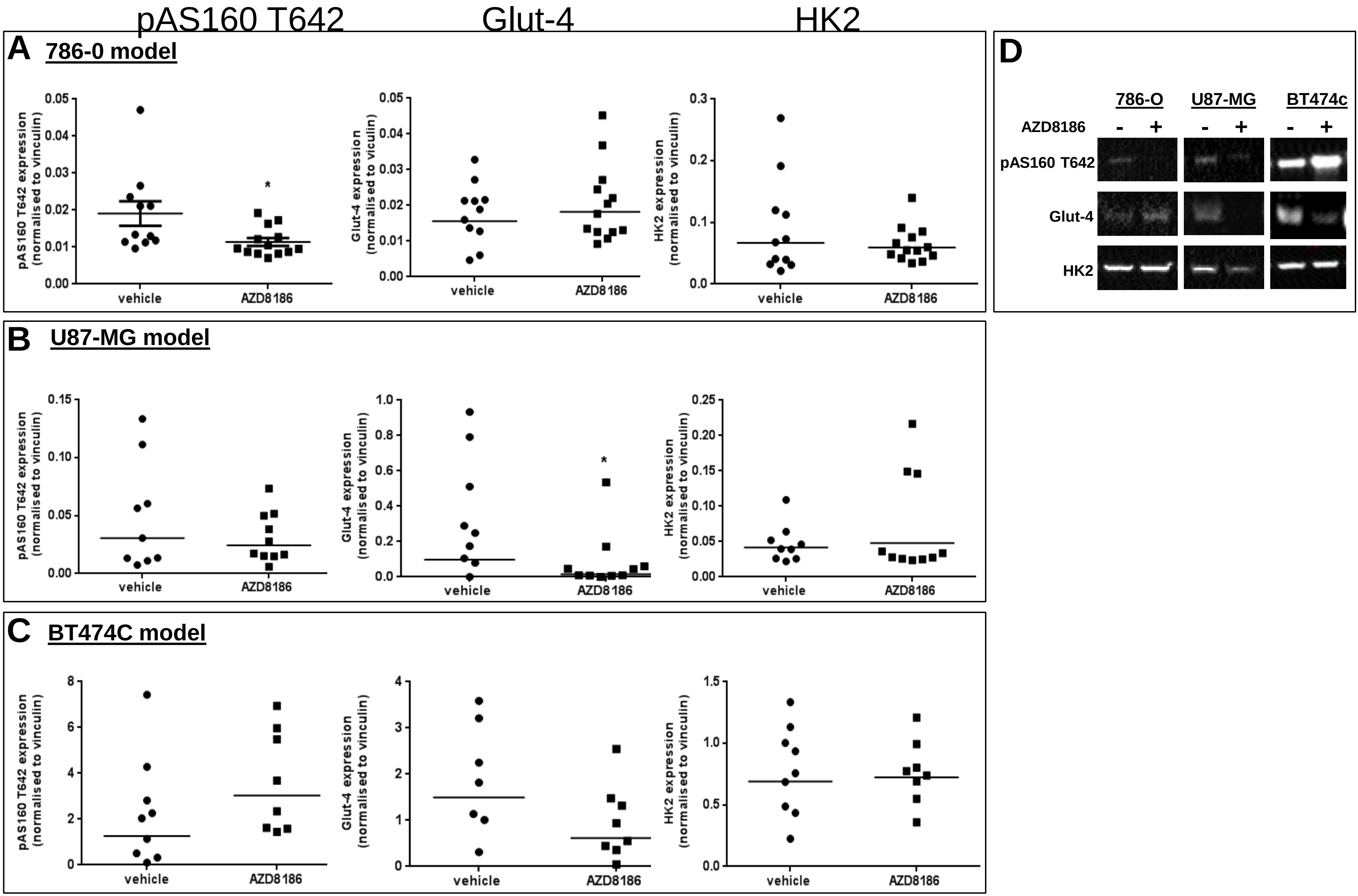

Supplementary Figure 2: A single dose of AZD8186 (50mg/kg) results in a non-uniform modulation of biomarkers associated with glucose uptake in different tumour models tested. Phosphorylation of AS160 (Thr642) and expression of Glut-4 and HK2: A) 786-0 model; B) U87-MG model; C) BT474C model. Western blot data is shown for individual animals and geomean indicated; n of at least 8 tumours/group. D) Example Western blots for each biomarker in each model without and with AZD8186 treatment.
