# Supplementary material for: The use of 18F-fluorodeoxyglucose positron emission tomography (18F-FDG PET) as a pathway-specific biomarker with AZD8186, a PI3Kβ/δ inhibitor
Source: EJNMMI Res. 2016 Aug 11;6:62. doi: 10.1186/s13550-016-0220-9 (PMC4980858; doi:10.1186/s13550-016-0220-9)
Supplement: Additional file 3: Figure S3. — A single dose of AZD8186 (50 mg/kg) results in only limited apoptotic cell death and upregulatedcellular stress in PTEN null models. Cleaved caspase-3 (apoptosis) and γH2AX Ser139 levels (DNA damage, stress response): A) 786-0 model; B) U87-MG model; C) BT474C model. Western blot data is shown for individual animals and geomeanindicated; n > 8/group. D). Example Western blots for each biomarker in each model without and with AZD8186 treatment. (PDF 166 kb) [file 13550_2016_220_MOESM3_ESM.pdf]

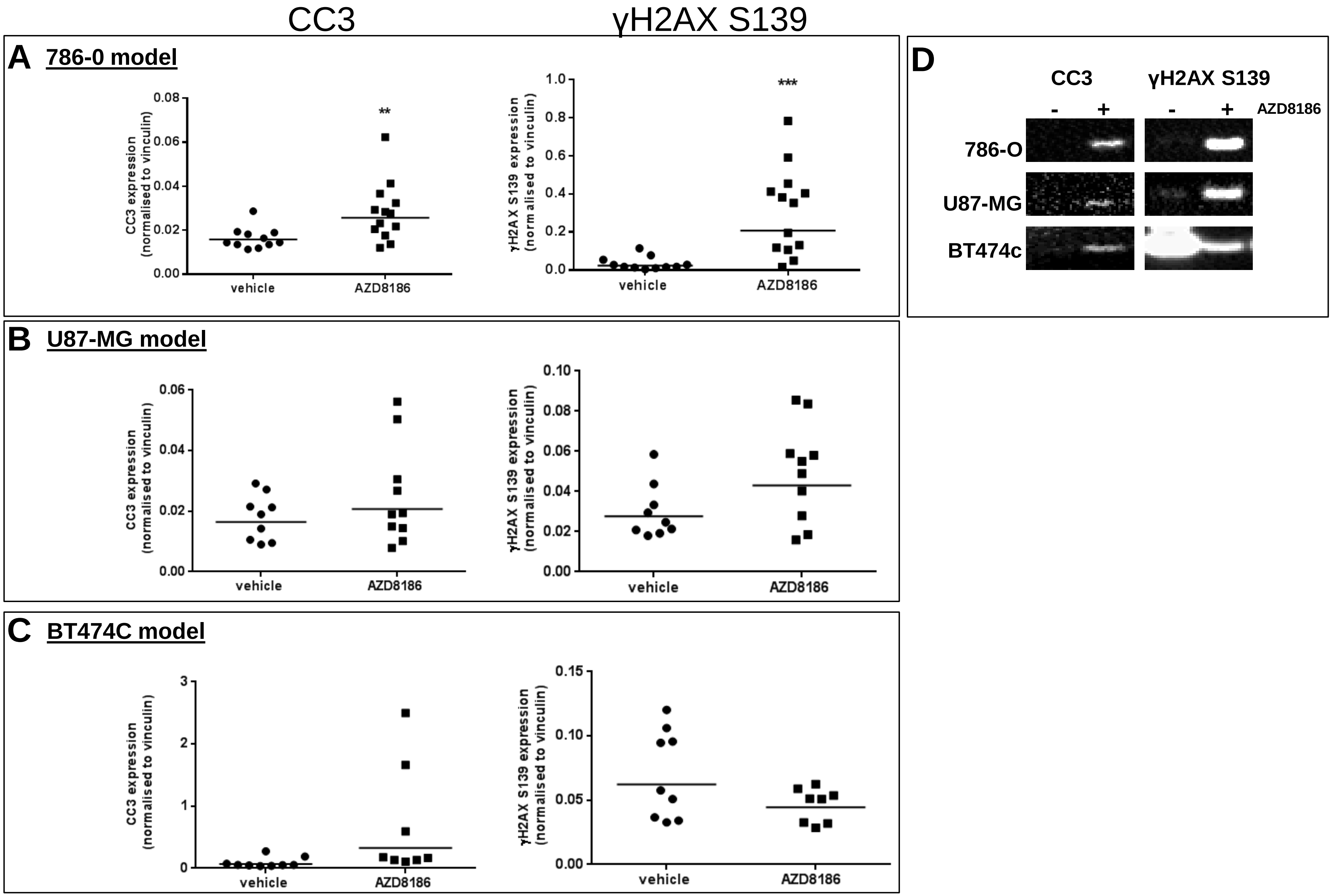

Supplementary Figure 3: A single dose of AZD8186 (50mg/kg) results in only limited apoptotic cell death and upregulated cellular stress in PTEN null models. Cleaved caspase-3 (apoptosis) and γH2AX Ser139 levels (DNA damage, stress response) A) 786-0 model: B) U87-MG model: C) BT474C model. Western blot data is shown for individual animals and geomean indicated; n>8/group. D). Example Western blots for each biomarker in each model without and with AZD8186 treatment.
